# Th17 Cell-Mediated Colitis Is Positively Regulated by Interferon Regulatory Factor 4 in a T Cell-*Extrinsic* Manner

**DOI:** 10.3389/fimmu.2020.590893

**Published:** 2021-01-29

**Authors:** Vera Buchele, Patrick Konein, Tina Vogler, Timo Kunert, Karin Enderle, Hanif Khan, Maike Büttner-Herold, Christian H. K. Lehmann, Lukas Amon, Stefan Wirtz, Diana Dudziak, Markus F. Neurath, Clemens Neufert, Kai Hildner

**Affiliations:** ^1^ Department of Medicine 1, University Hospital Erlangen, University of Erlangen-Nuremberg, Erlangen, Germany; ^2^ Deutsches Zentrum Immuntherapie (DZI), University Hospital Erlangen, University of Erlangen-Nuremberg, Erlangen, Germany; ^3^ Institute of Pathology, Department of Nephropathology, University Hospital Erlangen, Erlangen, Germany; ^4^ Laboratory of Dendritic Cell Biology, Department of Dermatology, University Hospital Erlangen, Friedrich-Alexander University of Erlangen-Nuremberg, Erlangen, Germany

**Keywords:** interferon regulatory factor 4 (IRF4), myeloid cells, Th17, intestinal inflammation, inflammatory bowel disease (IBD)

## Abstract

Inflammatory bowel diseases (IBDs) are characterized by chronic, inflammatory gastrointestinal lesions and often require life-long treatment with immunosuppressants and repetitive surgical interventions. Despite progress in respect to the characterization of molecular mechanisms *e.g.* exerted by TNF-alpha, currently clinically approved therapeutics fail to provide long-term disease control for most patients. The transcription factor interferon regulatory factor 4 (IRF4) has been shown to play important developmental as well as functional roles within multiple immune cells. In the context of colitis, a T cell-intrinsic role of IRF4 in driving immune-mediated gut pathology is established. Here, we conversely addressed the impact of IRF4 inactivation in non-T cells on T cell driven colitis *in vivo*. Employing the CD4^+^CD25^−^ naïve T cell transfer model, we found that T cells fail to elicit colitis in IRF4-deficient compared to IRF4-proficient *Rag1* ^−/−^ mice. Reduced colitis activity in the absence of IRF4 was accompanied by hampered T cell expansion both within the mesenteric lymph node (MLN) and colonic lamina propria (cLP). Furthermore, the influx of various myeloids, presumably inflammation-promoting cells was abrogated overall leading to a less disrupted intestinal barrier. Mechanistically, gene profiling experiments revealed a Th17 response dominated molecular expression signature in colon tissues of IRF4-proficient, colitic *Rag1* ^−/−^ but not in colitis-protected *Rag1* ^−/−^ *Irf4* ^−/−^ mice. Colitis mitigation in *Rag1* ^−/−^ *Irf4* ^−/−^ T cell recipients resulted in reduced frequencies and absolute numbers of IL-17a-producing T cell subsets in MLN and cLP possibly due to a regulation of conventional dendritic cell subset 2 (cDC2) known to impact Th17 differentiation. Together, extending the T cell-intrinsic role for IRF4 in the context of Th17 cell driven colitis, the provided data demonstrate a Th17-inducing and thereby colitis-promoting role of IRF4 through a T cell-extrinsic mechanism highlighting IRF4 as a putative molecular master switch among transcriptional regulators driving immune-mediated intestinal inflammation through both T cell-intrinsic and T cell-extrinsic mechanisms. Future studies need to further dissect IRF4 controlled pathways within distinct IRF4-expressing myeloid cell types, especially cDC2s, to elucidate the precise mechanisms accounting for hampered Th17 formation and, according to our data, the predominant mechanism of colitis protection in *Rag1* ^−/−^ *Irf4* ^−/−^ T cell receiving mice.

## Introduction

Inflammatory bowel diseases (IBDs) clinically manifest with chronic affections of the gastrointestinal tract that are assumed to result from an inadequate immune response while antigens against which the immune attack may be specifically directed have not been identified so far (1). Besides some early-onset cases affecting infants that are due to a rather genetically circumscribed mutation as *e.g*. IL-10 receptor gene, onset of disease usually occurs in young to middle-aged adults and is triggered by so far rather undisclosed environmental factors that however epidemiologically seem to be associated with western life style habits as *e.g.* type of nutrition (1–3). Among the diverse and complex pool of immune cell subpopulations found within the inflamed gut of IBD patients, based on many preclinical experimental data sets, T cells are assumed to play a major pathogenetic role in mediating intestinal tissue inflammation (4–6). In fact, interleukin 17a (IL-17a) producing T helper (Th17) cells are one of the most prevalent T cell subsets in the inflamed gut tissue, suggesting a critical contribution to the pathogenesis of IBD (7). However, failure in clinical studies examining the efficacy of antibody mediated IL-17a and IL-17R blockade in IBD was therefore unexpected and may indicate that pro-inflammatory effects of Th17 cells are not or at least not exclusively mediated by the cytokine IL-17a alone with the latter putatively exerting rather barrier-protective effects in this context given the observation of disease aggravation following IL-17a neutralization in some patients (8, 9). Regardless, data on the biology and function of IL-23 in IBD argue for the overall colitogenic rather than inflammation-reducing nature of Th17 cells given the fact that IL-23 has been revealed to be one, if not the most important cytokine acting upstream of Th17 cells providing crucial signals for their survival and proliferation (10–13). Interestingly, recently IL-23 which expression is regularly upregulated in IBD tissues was suggested to be critically involved in driving alternative immune pathways specifically active in patients suffering from an anti-TNF-alpha blockade resistant disease (14). Overall, in addition to strategies that specifically block gut homing mediating molecules, IL-23 represents together with TNF-alpha one of the few already therapeutically established biological targets in clinical management of IBD further strengthening the case for the central pathogenicity of IL-23R^+^ Th17 cells in the context of IBD.

Antigen-presenting cells (APCs) have been identified and characterized to be critical instructors and modulators of both pro- and anti-inflammatory T cell responses *in vivo* (15–18). In addition to providing co-stimulatory or -inhibitory signals, APCs do so largely by expressing and releasing cytokines as *e.g.* IL-12, IL-23, or TGF-*ß* all known to be crucial upstream regulators and promoters of pro-inflammatory or regulatory T cell differentiation programs (16, 17, 19, 20). T cells themselves are unable to express inflammation-promoting cytokines like IL-23 and IL-12. Hence, *e.g*. IL-23 is largely provided by APC as *e.g.* dendritic cells and monocytes with the latter shown to have the ability to differentiate into inflammatory dendritic cells in the context of mucosal inflammation (16, 19, 21–23). Dendritic cells are subdivided into conventional (cDCs) and plasmacytoid dendritic cells (pDCs). Based on the developmental dependence on specific transcriptional regulators and critical functional differences in respect to their differential abilities to induce and promote certain types of T cell responses, cDCs can be further differentiated into two major subsets, cDC1 and cDC2 (15, 24, 25). cDC1s have been shown to be particularly critical for the induction of anti-viral and anti-tumor CD8^+^ T cell responses in part by the preferential ability to release IL-12 and cDC1 development is dependent on the transcription factor axis IRF8/BATF3/ID2 (26–29). In contrast, development and functionality of cDC2 are largely dependent on the transcription factor IRF4 (16, 30, 31). Interestingly, cDC2s have been shown to represent a critical source for IL-23 expression *in vivo* suggesting that especially IRF4 dependent cDC2s might represent critical APC driving Th17 cell responses *in vivo* as *e.g.* in the context of colitis (15, 16, 19). While the T cell-intrinsic function of IRF4 in regard to its contribution to the manifestation of colitis has been thoroughly evaluated (32), the question whether IRF4 expressed by non-T cells is involved in the colitis manifestation and more specifically in the orchestration of the colitogenic T cell responses and if so in what way has not been studied in great detail so far. Hence, here we assessed the T cell-extrinsic role of IRF4 for the course of acute T cell driven intestinal inflammation employing the widely accepted CD4^+^CD25^−^ naïve T cell transfer model system (33, 34). We found that IRF4 expressed in non-T cells is indispensable for the clinical, endoscopic, and histopathological colitis manifestation. Moreover, IRF4 deficiency within mice receiving IRF4-expressing T cells resulted in a decreased recovery rate of transferred T cells both in the draining mesenteric lymph node and the colonic lamina propria. Most importantly, formation of colitogenic T cells subsets and here foremost IL-17a expressing Th17 cell subsets were severely hampered in the absence of T cell-extrinsic IRF4 expression. Given its established, cell-intrinsic role during the regulation of Th17 fate decision and cDC2 development and function with the latter known to promote Th17 immune responses, IRF4 overall emerges as a key transcriptional regulator globally promoting Th17 cell driven gut inflammation.

## Materials and Methods

### Mice

C57Bl/6 mice were purchased from Janvier Labs, and congenic CD45.1/Ly5.1 B6.SJL-PtrprcaPepcb/BoyCrl mice and B6.PL-*Thy1^a^*/CyJ mice were purchased from Charles River Laboratories and The Jackson Laboratory, respectively. B6.129S7-Rag1tm1Mom/J (termed *Rag1* ^−/−^ mice) and B6.129P2-*Irf4^tm1Mak^*/J (termed *Irf4* ^−/−^ mice) were purchased from The Jackson Laboratory and intercrossed to generate *Rag1* ^−/−^ *Irf4* ^−/−^ mice. Mice were maintained under specific pathogen-free conditions. Mice at 8 to 16 weeks of age were used. This study was carried out in accordance with the recommendations of the government of Lower Franconia in Bavaria, Germany. The protocol was approved by the government of Lower Franconia in Bavaria, Germany.

### T Cell Transfer Colitis Model

Splenocytes were isolated from wildtype donor mice (C57Bl/6, CD45.1/Ly5.1 B6.SJL-PtrprcaPepcb/BoyCrl or B6.PL-*Thy1^a^*/CyJ). For this purpose, the spleen was mashed through a 40 µm cell strainer and red blood cells were lysed with ACK buffer (0.15 M NH_4_Cl, 1 M KHCO_3,_ 0.8 M Na_2_EDTA; pH7.2). Naïve (CD4^+^CD25^−^) splenic T cells were isolated from spleen cell suspensions by two consecutive magnetic cell separation steps with the CD4^+^ T cell isolation kit followed by the CD25 Microbead kit (Miltenyi Biotec) according to the manufacturer’s instructions. Purity of cell isolates was confirmed by flow cytometry. To induce transfer colitis, 1 × 10^6^ naïve T cells were injected i.p. into recipient mice. *Rag1* ^−/−^ *Irf4* ^−/−^ mice were used as IRF4-deficient recipients. Either *Rag1* ^−/−^ *Irf4* ^+/+^ or *Rag1* ^−/−^ *Irf4* ^+/−^ mice were used as IRF4-proficient T cell recipients due to a virtually indistinguishable colitis phenotype of wildtype (*Irf4* ^+/+^) and IRF4 heterozygous (*Irf^-^* ^+/−^) mice, and this group is further referred in the manuscript as *Rag1* ^−/−^ mice.

### Mini-Endoscopy of Mice

Mucosal inflammation of the colon after T cell transfer was assessed macroscopically by colonoscopy using an image 1™ S3 mini-endoscope (Karl Storz) as previously described (35). For this purpose, mice were anesthetized and inflammation of the colon was assessed using a modified murine endoscopic index of colitis severity (MEICS) based on four parameters: thickening of the bowel wall, changes of the vascularity, granularity of the mucosal surface and stool consistency. Every parameter was scored from zero for no colitis to three for massive inflammation adding up to a maximal score of 12 as previously described (36).

### Histopathological Analysis

Samples of the distal colon were rinsed with phosphate-buffered saline (PBS; Sigma-Aldrich) and fixed in 4.5% formaldehyde (Carl Roth) overnight. For histopathological analysis, sections of paraffin-embedded colon tissue were stained with hematoxylin and eosin (H&E), and inflammation was assessed by a pathologist in a blinded manner based on a slightly modified scoring system described by Erben et al. (37). Slides were analyzed using a Zeiss Axio Imager.A1 microscope and measurements were performed in micrographs taken with a Zeiss AxioCam MRc camera and Zeiss AxioVision (4.9.1.SP2) software. Briefly, histopathological changes were scored for four criteria: inflammatory density (0 = no/minimal, 1 <10%, 2 = 10–25%, 3 = 26–50%, 4 >50%), hyperplasia (0 <200 µm crypt length, 1 = 200–299 µm, 2 = 300–399 µm, 3 >400 µm), goblet cell loss (0 <20%, 1 = 21–35%, 2 = 36–50%, 3 >50%) and crypt abscesses (0 = none, 1 <one, 2 = one to two, 3 = three or more per quadrant in a circumferential colonic section). In addition, the location of inflammation was assessed (1 = only mucosa, 2 = extending into the submucosa, 3 = transmural), the crypt loss was scored from 0 to 2 (0 = none, 1 = one, 2 = two or more neighboring crypts lost), and the absence or presence of erosion, ulceration and irregular crypts was scored with 0 or 1, respectively. Scores of each criterion were summed up leading to a maximum total histology score of 21.

### Immunohistochemistry

Samples of the distal part of the colon were frozen and embedded in Optimal Cutting Temperature compound (Sakura). Frozen tissue sections were fixed in 2% paraformaldehyde for 20 min. To prevent unspecific binding, sections were blocked for 1 h with blocking buffer consisting of 10% fetal calf serum (FCS; Pan-Biotech) and 1% bovine serum albumin (Sigma-Aldrich). Sections were double-stained with anti-F4/80-Alexa Fluor 488 (BM8; BioLegend) and anti-CD4-Alexa Fluor 647 (GK1.5; BioLegend) or rabbit anti-MPO (polyclonal; Abcam) in blocking buffer overnight at 4°C. For MPO staining, sections were additionally incubated with donkey anti-rabbit-IgG-Alexa Fluor 647 (Poly4064; BioLegend) for 2 h at room temperature in blocking buffer. Nuclei were counterstained with Hoechst 33342 (Life Technologies). Images were recorded using the confocal microscope Leica TCS SP5II with Leica LasX software.

### Isolation of Colonic Lamina Propria Cells, Splenocytes, and Mesenteric Lymph Node Cells

Colonic lamina propria (cLP) cells were isolated by enzymatic digestion as described before (38). Briefly, the colon was rinsed with PBS to remove intestinal content, opened longitudinally, and cut into small pieces. After washing colonic pieces twice with Hanks’ Balanced Salt solution (HBSS; Sigma-Aldrich) supplemented with EDTA (0.5 mM), intestinal tissue was digested in HBSS containing DNase I (0.25 mg/ml), collagenase D (0.5 mg/ml), dispase II (3 Units/ml), and 5% FCS. Following filtering through a 40 µm cell strainer (Falcon) and washing of the digested tissue with PBS, cLP cells were enriched with density gradient centrifugation where 80% Percoll was overlaid with cells resuspended in 40% Percoll (GE Healthcare). cLP cells were washed with RPMI supplemented with 10% FCS prior to further analysis. Splenocytes and mesenteric lymph node (MLN) cells were isolated by enzymatic digestion of chopped tissues with DNase I (30 U/ml), collagenase B (0.25 mg/ml) in DMEM high glucose (Gibco) supplemented with 10% FCS for 1 h at 37°C. Cells were filtered through a 40 µm cell strainer and washed with PBS. Red blood cells were lysed with ACK buffer. All enzymes were purchased from Sigma-Aldrich.

### Flow Cytometry Analysis

For the analysis of cell surface markers, isolated cells were stained with fluorochrome-conjugated antibodies dissolved in staining buffer (3% FCS in PBS) for 20 min at 4°C in the dark before analysis. For staining of intranuclear proteins, the FOXP3 Fix/Perm buffer set (BioLegend) was used according to the manufacturer’s instructions. Briefly, following staining of cell surface markers, cells were fixed in fixation/permeabilization working solution for 40 min at 4°C in the dark before permeabilization and staining in permeabilization buffer for 40 min at 4°C in the dark. For intracellular cytokine staining, isolated cells (1 × 10^6^/ml) were cultured in cell culture medium [DMEM high glucose containing 10% FCS, 100 U/ml penicillin/0.1 mg/ml streptomycin (Sigma-Aldrich), 1% non-essential amino acids (Sigma-Aldrich), 1% L-glutamine (Sigma-Aldrich), 1 mM sodium pyruvate (Sigma-Aldrich) and 0.5 mM *β*-mercaptoethanol (Gibco)] in the presence of 50 ng/ml phorbol 12-myristate 13-acetate (Sigma-Aldrich) and 1 µM ionomycin (Sigma-Aldrich) for 4 h at 37°C. 1 µg/ml brefeldin A (Sigma-Aldrich) was added for the last 3 h of culture. Thereafter, cells were stained for surface markers. Intracellular cytokine staining was performed as described previously (39). In brief, following fixation with 2% formaldehyde for 15 min, cells were permeabilized with 0.05% saponin (Sigma-Aldrich) in staining buffer. Intracellular cytokines were stained for 30 min at 4°C in the dark using fluorochrome-labeled antibodies dissolved in 0.5% saponin in staining buffer. To identify and exclude dead cells DAPI (Sigma-Aldrich) or the LIVE/DEAD Fixable Aqua Dead Cell Stain Kit (Life Technologies) was used according to the manufacturer’s instructions. Stained cells were measured in staining buffer on a FACSFortessa II (BD Biosciences) flow cytometer, and data were analyzed using FlowJo 10.7.1 software (Tree Star Inc). Cell aggregates were excluded from analysis using forward scatter-area *versus* forward scatter-height scatterplots. The following antibodies were used: anti-Ly6C (HK1.4), anti-Ly6G (IA8), anti-F4/80 (BM8), anti-CD3*ϵ* (17A2), anti-CD4 (GK1.5), anti-GM-CSF (MP1-22E9), anti-IL-17a (TC11-18H10.1), anti-IFN-*γ* (XMG1.2); anti-CD45.2 (104), anti-I-A/I-E (M5/114.15.2), anti-CD11c (N418), anti-CD103 (2E7), anti-CD11b (M1/70), anti-IRF-4 (IRF4.3E4), anti-CD172 (A7R34), anti-T-bet (4B10), anti-XCR1 (ZET), streptavidin-Brilliant-Violet 421 from BioLegend, anti-CD11b (M1/70), anti-ROR*γ*t (Q31-378), anti-I-A/I-E (M5/114.15.2), anti-CD45 (30F11), anti-CD19 (1D3), anti-CD3*ϵ* (17A2), anti-NK1.1 (PK136), anti-CD11c (HL3) from BD Biosciences, Anti-CD172a (P84) from Life Technologies, anti-Thy1.2 (30-H12), anti-GATA3 (REA174) and a custom made biotinylated lineage antibody cocktail (anti-B220, anti-CD3, anti-CD5, anti-CD11b, anti-GR1, anti-NK1.1, anti-SiglecF, anti-Ter119) from Miltenyi Biotec.

### Quantitative Real-Time Polymerase Chain Reaction

Following RNA isolation from a sample of distal colonic tissue using the NucleoSpin RNA isolation kit (Macherey Nagel), cDNA of 1 µg RNA was reverse transcribed using iScript cDNA Synthesis kit (Bio-Rad Laboratories). RNA quality and concentration were measured with a Nanodrop ND-1000 (Thermo Fisher Scientific). Gene expression was measured by quantitative Real-Time polymerase chain reaction (qPCR) using iQ SYBR Green Supermix (Bio-Rad Laboratories) and the following primers (MWG Eurofins): *Hprt* forward 5′-TGG ATA CAG GCC AGA CTT TGT T-3′, reverse 5′-CAG ATT CAA CTT GCG CTC ATC-3′, *Ifng* forward 5′-ATC TGG AGG AAC TGG CAA AA-3′, reverse 5′-TGA GCT CAT TGA ATG CTT GG-3′, *Csf2* forward 5′-ATC AAA GAA GCC CTG AAC CT-3′, reverse 5′-GTG TTT CAC AGT CCG TTT CC-3′, *Tnf* forward 5′-CTT GTG GCA GGG GCC ACC AC-3′, reverse 5′-CCA TGC CGT TGG CCA GGA GG-3′, *Il-17a* forward 5′-GCT CCA GAA GGC CCT CAG A-3′, reverse 5′-AGC TTT CCC TCC GCA TTG A-3′, *Il1b* forward 5′-GTG ACG TTC CCA TTA GAC AA-3′, reverse 5′-TAT TTT GTC GTT GCT TGG TT-3′, *Rorc* forward 5′-CCG CTG AGA GGG CTT CAC-3′, reverse 5′-TGC AGG AGT AGG CCA CAT TAC A-3′, *Il23r* forward 5′-CAC AAC AAC TAC ACG TCC AT-3′, reverse 5′-TAC CAG TTT CTT GAC ATC GC-3′, *Batf* forward 5′-GGA AGA TTA GAA CCA TGC CTC-3′, reverse 5′-CCA GGT GAA GGG TGT CGG-3′, *Il6* forward 5′-CCG GAG AGG AGA CTT CAC AG-3′, reverse 5′-TTC TGC AAG TGC ATC ATC GT-3′, *Il12a* forward 5′-ACA GGG TGA TGG GCT ATC TG-3′, reverse 5′-GGG GAG ATG AGA TGT GAT GG-3′, *Il23r* forward 5′-CAC AAC AAC TAC ACG TCC AT-3′, reverse 5′-TAC CAG TTT CTT GAC ATC GC-3′. Primer sets for *Tbx21* and *Il23a* were purchased from QIAGEN. Samples were run on a CFX Connect and CFX96 Real-Time PCR detection system (Bio-Rad Laboratories). Data were analyzed with CFX Manager v3.1 (Bio-Rad Laboratories). Expression levels of target genes for each sample were normalized relative to the reference gene *Hprt*. Relative gene expression levels were calculated with the 2(^−ΔΔCt^) method. Mean gene expression levels detected in *Rag1* ^−/−^ mice were arbitrarily set to one and gene expression levels of *Rag1* ^−/−^ *Irf4* ^−/−^ mice were calculated and displayed in relation to the normalized *Rag1* ^−^ *^/^* ^−^ mice.

### Statistical Analysis

Unpaired Student’s *t* test was used for comparison of means of two datasets. *p <0.05 was considered to be significant. Statistical analysis was performed with Graphpad Prism 8.3.0 software.

## Results

### *Rag1* ^−/−^ *Irf4* ^−/−^ Mice Fail to Develop Colitis Upon Transfer of Naïve CD4^+^ T Cells

**Figure 1:**
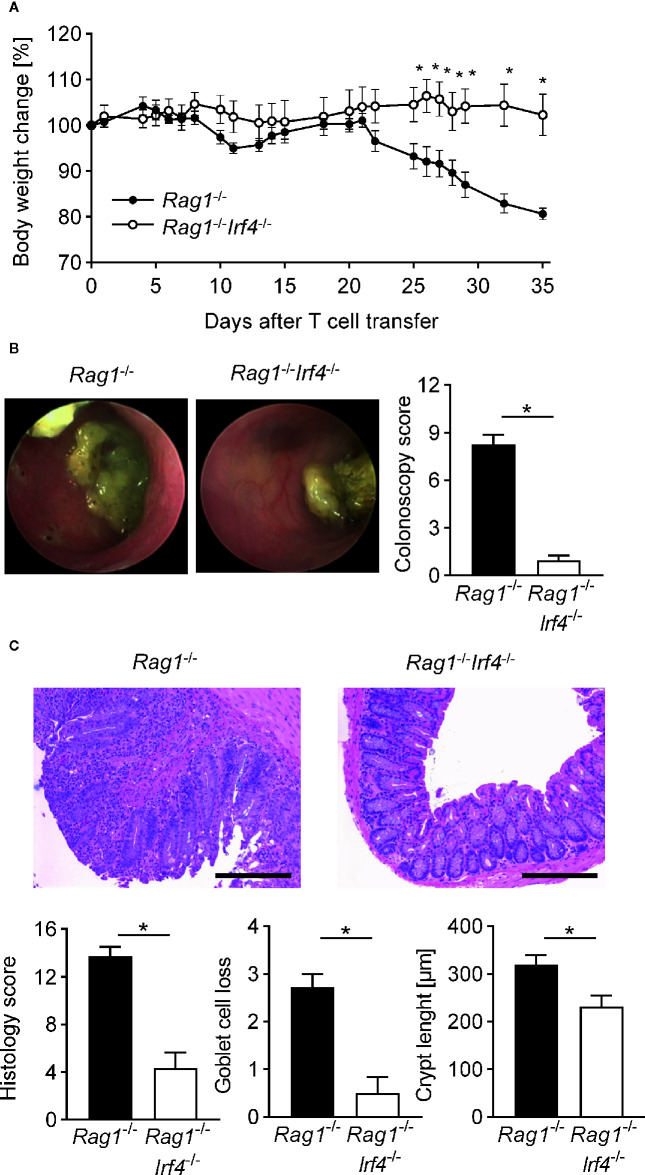
Inactivation of IRF4 in non-T cells abrogates clinical, endoscopic, and histopathological signs of colitis. At day 0 *Rag1* ^−/−^ and *Rag1* ^−^ *^/^* ^−^ *Irf4* ^−/−^ mice were injected i.p. with 1 × 10^6^ naïve (CD4^+^CD25^−^) T cells. **(A)** Percentage body weight changes compared to the original body weight at day 0 were assessed over time. A representative course of body weight changes per experimental group from one of four experiments is shown (*Rag1* ^−/−^ n = 6; *Rag1* ^−^ *^/^* ^−^ *Irf4* ^−/−^ n = 5). **(B)** Colitis severity was analyzed by colonoscopy when T cell recipient *Rag1* ^−^ *^/^* ^−^ mice showed sustained weight loss of more than 10% of their initial body weight for at least one week (four to five weeks after T cell transfer). Colonoscopy score of three independent experiments and representative endoscopic images for every experimental group are shown. *Rag1* ^−/−^ and *Rag1* ^−^ *^/^* ^−^ *Irf4* ^−/−^ mice were sacrificed when colitis was established in T cell recipient *Rag1* ^−/−^ mice identified by weight loss and colonic inflammation *via* colonoscopy (five to six weeks after T cell transfer). **(C)** Histopathological scoring of the inflammation present in the distal colon. One representative H&E-stained histopathological cross-section of the distal colon per group is shown. Scale bars: 100 µm. **(B**, **C)** Data are combined from three individual experiments (*Rag1* ^−/−^ n = 11; *Rag1* ^−^ *^/^* ^−^ *Irf4* ^−/−^ n = 12). Data were analyzed by Student’s *t* test and are shown as mean ± SEM. *p <0.05.

Previously, we and others have reported that in the T cell transfer colitis model inflammatory manifestations within the gut are strongly dependent on T cell-intrinsic expression of transcription factors proven to be indispensable for the differentiation of Th17 cells *in vitro* and *in vivo* (32, 40–42). In line with that, T cell restricted IRF4-deficiency severely compromised T cells’ ability to induce colitis (32). Conversely, however, the selective role IRF4 expressed by non-T cells exerts in the immune pathogenesis of colitis remains largely unresolved. To decipher the unknown impact of IRF4 in this setting, we sought to create a genetic model in which IRF4 deficiency is restricted to the non-T cell compartment while T cells remain fully competent, *i.e.* T cells with preserved IRF4 expression abilities. In this experimental set-up, any kind of regulation of observed T cell colitogenicity would be secondary to the T cell-extrinsic IRF4 deficiency, *i.e.* clearly attributable to the altered functionality of non-T cells in the absence of IRF4. Thereby, this data set was suitable to help define the T cell-extrinsic role of IRF4 in the context of colitis. To achieve this goal, we crossed IRF4 germline deficient mice onto the Rag1-deficient background and elicited colitis in these mice upon naïve CD4^+^ T cell transfer into *Rag1* ^−/−^ *Irf4* ^−/−^, *Rag1* ^−/−^ *Irf4* ^+/−^ or *Rag1* ^−/−^ *Irf4* ^+/+^ mice with the last two groups serving as IRF4-sufficient control animals (termed *Rag1* ^−/−^ mice) (33, 34, 43). Strikingly, as documented by the dynamic changes of the body weight curves over time, after 7–10 days, *i.e.* upon completion of priming and start of substantial expansion of transferred CD4^+^ T cells, IRF4-proficient recipient mice started to lose initially subtly but progressively increasing weight compared to IRF4-deficient *Rag1* ^−/−^ mice with a clear separation of both curves at the beginning of the fourth week after experimental start (**Figure 1A**).

To assess whether the observed weight loss is accompanied by or even due to an acute immune-mediated affection of the colonic barrier, we performed mini-endoscopic evaluation of the distal colon of both IRF4-sufficient and -deficient *Rag1* ^−/−^ mice *in vivo* prior sacrificing mice (**Figure 1B**). As displayed in **Figure 1B**, at this time point colonoscopy showed that IRF4-competent mice suffered from severe colitis in respect to all evaluated categories adding up to a mean sum score of around 8. In contrast, T cell receiving *Rag1* ^−/−^ mice deficient in IRF4 virtually lacked macroscopic signs of colitis evidenced by a sum score of <3 with a score of ≥3 representing the empiric cut-off value for mice suffering from clinically meaningful, endoscopic signs of colitis. To further validate the macroscopic results, we performed thorough histopathological studies by evaluating cross-sections derived from the distal colon, *i.e.* areas matching the mini-endoscopically assessed region (**Figure 1C**). Here, histopathological scoring and grading revealed that both goblet cell loss and crypt length increase commonly observed following severe forms of intestinal inflammation were decreased in *Rag1* ^−/−^ *Irf4* ^−/−^ T cell receiving mice. Overall, histopathologically assessed parameters summarized in the histology score underscore and further extend the results obtained during endoscopic evaluation that *Rag1* ^−/−^ mice lacking IRF4 expression are largely protected from T cell mediated colon inflammation (**Figure 1C**). Overall data presented so far unequivocally demonstrate that IRF4 expression within non-T cells is indispensable for T cell mediated transfer colitis in *Rag1* ^−/−^ recipient mice based on both clinical, endoscopic, and histopathological scoring results.

### Colitis Protection of IRF4 Deficient *Rag1* ^−/−^ Mice Is Associated With a Reduced Expansion of Transferred T Cells and Diminished Recruitment of Inflammatory Mononuclear Cells Into the Colon

**Figure 2:**
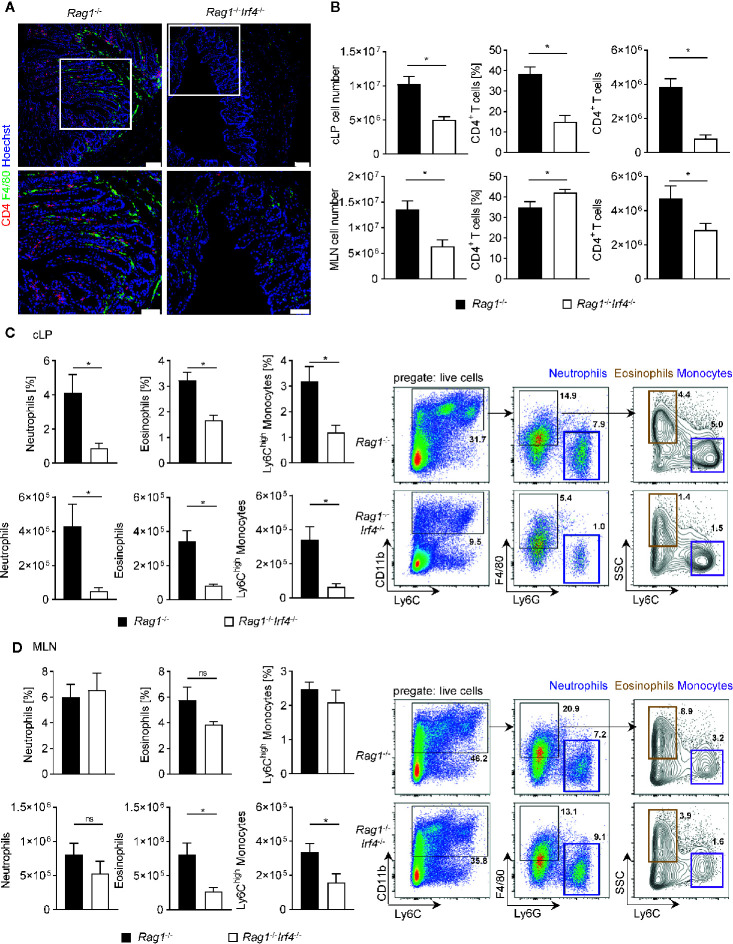
Hampered expansion and influx of both recipient-derived myeloid and donor-derived T cells in T cell receiving *Rag1* ^−/−^ mice deficient in IRF4. *Rag1* ^−/−^ and *Rag1* ^−^ *^/^* ^−^ *Irf4* ^−/−^ mice were injected i.p. with 1 × 10^6^ naïve (CD4^+^CD25^−^) T cells. When colitis was established in *Rag1* ^−/−^ mice five to six weeks after T cell transfer, both *Rag1* ^−/−^ and *Rag1* ^−^ *^/^* ^−^ *Irf4* ^−/−^ mice were sacrificed, and the influx of T cells and inflammatory myeloid cells into the colon and mesenteric lymph node (MLN) was analyzed. **(A)** Representative immunofluorescence staining of F4/80^+^ and CD4^+^ cells in colonic cross sections at day 39 after T cell transfer. Upper panel: overview picture of the colonic tissue (scale bars = 75 µm); lower panel: higher magnification of the white boxed area within the colonic tissue of the upper panel (scale bars = 50 µm). **(B)** Absolute number of colonic lamina propria (cLP) and MLN was determined (left column). Frequencies (middle column) and absolute numbers (right column) of CD4^+^ T cells (CD3^+^CD4^+^) within the total live immune cell pool were analysed by flow cytometry. In addition relative fraction (upper panel) and absolute cellularity (lower panel) of neutrophils (CD11b^+^F4/80^−^Ly6G^+^Ly6C^+^), eosinophils (CD11b^+^F4/80^+^Ly6G^-^SSC^high^Ly6C^−^) and inflammatory Ly6C^high^ monocytes (CD11b^+^F4/80^+^Ly6G^-^SSC^low^Ly6C^high^) within the total live immune cell pool of cLP **(C)** and MLN **(D)** cells were analyzed by flow cytometry. One representative flow cytometry plot is shown for every experimental group and illustrates the gating strategy for the indicated cell populations. Frequencies of cells in each sub-gate are calculated as a percentage of live cells. Data are combined from two individual experiments (*Rag1* ^−/−^ n = 7; *Rag1* ^−^ *^/^* ^−^ *Irf4* ^−/−^ n = 8). Data were analyzed by Student’s *t* test and are shown as mean ± SEM. ns, not significant. *p < 0.05.

To further deconstruct the mechanism underlying reduced manifestation of intestinal inflammation in *Rag1* ^−/−^ *Irf4* ^−/−^ mice when compared to controls, we next focused on the quantitative and qualitative regulation of the cellular composition within the inflamed gut. First, we performed multi-color immunofluorescence (IF) staining of distal colon cross-sections matching previously histopathologically assessed areas (**Figure 2A**). We readily observed a striking reduction of the total immune cell infiltration in *Rag1* ^−/−^ mice lacking IRF4 expression. In line with our IF data, cell number calculations of isolated colonic lamina propria mononuclear cell preparations confirmed a significant reduction of total cLP cells in mice lacking IRF4 compared to IRF4-sufficient controls **(** **Figure 2B** **)**. Furthermore IF imaging suggested that transferred CD4^+^ T cells are less abundant in *Rag1* ^−/−^ *Irf4* ^−/−^ mice compared to controls, and this finding was further substantiated by additional flow cytometry-based enumeration of CD4^+^ T cells within the total live cLP-derived immune cell pool (**Figures 2A, B** **)**. To further dissect whether this observation results from a general reduction of T cell activation/proliferation or is rather due to a gut homing phenotype due to hampered migratory properties of the T cells in the absence of IRF4 expression in non-T cells, we determined both the total cellularity and relative fraction of T cells within the mesenteric lymph node (MLN) cells by flow cytometry. Interestingly, similar to the cLP compartment, total numbers of MLN residing immune cells were reduced in *Rag1* ^−/−^ *Irf4* ^−/−^ mice compared to controls (**Figure 2B**). Also, the absolute numbers of CD4^+^ T cells were significantly diminished within the MLN suggesting that at least to a certain degree hampered systemic T cell activation and expansion may account for the lack of a colitis-mediating T cell pool in the absence of T cell-extrinsic IRF4 expression. However, despite that finding, the fraction of CD4^+^ T cells within the MLN residing immune cell pool was even relatively increased compared to control mice (**Figure 2B**). Hence, hampered efflux of CD4^+^ T cells from the MLN due to incomplete priming and/or imprinting of gut homing properties may be also contributing to the reduced representation of colitogenic T cells in the cLP fraction.

Intestinal myeloid cells have been described to play a dichotomic, context-dependent role under homeostatic and inflammatory conditions by exerting both anti-inflammatory as well as pro-inflammatory functions (22, 23, 44). Especially blood-derived monocytes are recruited to the gut through signal cascades initially set in motion by the intestinal immigration of cytokine releasing effector T cells where they were shown to at least partially convert into cells displaying a Ly6C^hi^ inflammatory phenotype (45–47). In fact, IF studies exploring the expression pattern of the surface glycoprotein and myeloid marker F4/80 revealed that colonic tissues of T cell receiving *Rag1* ^−/−^ *Irf4* ^−/−^ mice contained visibly reduced F4/80 expressing cells compared to controls contrasting the virtually indistinguishable pattern of F4/80^+^ and/or MPO^+^ cells resp. in the absence of inflammation between IRF4 proficient *vs.* deficient mice (**Figure 2A**, **Supplementary Figure 1A**). Further resolution in regard to the cellular composition of the F4/80^+^ cells was provided by additional in-depth flow cytometry analyses of cLP cell fractions. With the help of the shown gating strategy starting of CD11b^+^ cells, we are able to distinguish F4/80^+^Ly6G^neg^SSC^low^Ly6C^high^ inflammatory monocytes from F4/80^+^Ly6G^neg^SSC^low^Ly6C^neg^ eosinophils and F4/80^neg^Ly6C^+^Ly6G^+^ neutrophils (**Figure 2C**). Employing this gating approach, we readily found that the fraction of CD11b^+^ cells is dramatically increased within the pool of live cLP cells derived from T cell treated *Rag1* ^−/−^ mice with intact IRF4 expression compared to IRF4-deficient mice (**Figure 2C**). Moreover, we found that mononuclear cell isolates from the cLP compartment contained both absolutely and relatively diminished fractions of both neutrophils, eosinophils and presumably inflammatory Ly6C^high^ monocytes in *Rag1* ^−/−^ mice lacking IRF4 expression compared to IRF4-proficient controls (**Figure 2C**). In contrast, neither absolute nor relative IRF4-dependent regulation of either cell population prior T cell transfer, *i.e.* non-inflamed mice was detectable (**Supplementary Figure 1B**). Finally, similar to our studies on the T cell phenotype shown above in **Figures 2A, B**, we compared the composition of the MLN resident myeloid cell compartment in IRF4-sufficient *Rag1* ^−/−^ mice to *Rag1* ^−/−^ *Irf4* ^−/−^ mice prior and after T cell transfer. We could not detect any relative or absolute IRF4-dependent difference within granulocyte subsets and Ly6C^high^ monocytes in the absence of T cell induced colitis (**Supplementary Figure 1C**). Moreover, however contrasting our studies on the cLP compartment, the relative fractions of MLN residing neutrophils, eosinophils, and inflammatory monocytes were not increased in IRF4-competent compared to IRF4-deficient *Rag1* ^−/−^ mice after T cell transfer (**Figure 2D**). In addition, absolute numbers of neutrophils were virtually indistinguishable between T cell receiving *Rag1* ^−/−^ mice irrespective of their ability to express IRF4. However, in contrast, both absolute eosinophil as well as Ly6C^high^ monocyte counts were significantly reduced in MLN of *Rag1* ^−/−^ *Irf4* ^−/−^ mice compared to controls (**Figure 2D**). In summary, our data so far suggest that T cell-extrinsic inactivation of IRF4 leads to hampered T cell expansion both in MLN and cLP, while defective MLN exiting and/or gut homing abilities might add to the overall reduced recovery of putatively colitogenic cLP T cells. Furthermore, recruitment and/or local, *i.e.* intestinal expansion of neutrophils, eosinophils and Ly6C^high^ monocytes are severely compromised upon T cell transfer in *Rag1* ^−/−^ mice lacking IRF4 expression compared to IRF4 competent controls.

### IRF4 Induces a Colonic Th17 Gene Expression Signature in T Cell-Driven Colitis

**Figure 3:**
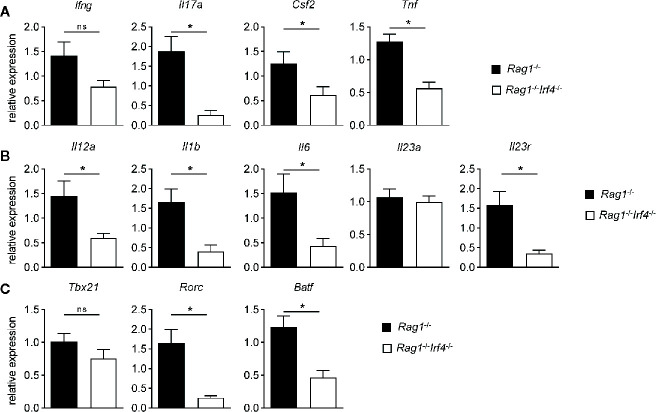
IRF4 drives a pro-inflammatory, Th17 cell differentiation promoting and putatively presence of functional Th17 cells increasing molecular gene expression signature in colitis tissues in a T cell-extrinsic manner. *Rag1* ^−/−^ and *Rag1* ^−^ *^/^* ^−^ *Irf4* ^−/−^ mice were injected i.p. with 1 × 10^6^ naïve (CD4^+^CD25^−^) T cells. When colitis was established in *Rag1* ^−/−^ mice five to six weeks after T cell transfer, both *Rag1* ^−/−^ and *Rag1* ^−^ *^/^* ^−^ *Irf4* ^−/−^ mice were sacrificed. Gene expression levels of **(A)** *Ifng*, *Il17a*, *Csf2*, *Tnf*, **(B)** *Il12a*, *Il1b*, *Il6*, *Il23a*, *Il23r*, **(C)** *Tbx21*, *Rorc*, and *Batf* transcripts within colonic tissue were analyzed and quantitated by qPCR. Mean of gene expression levels detected in colonic tissues of *Rag1* ^−/−^ mice was arbitrarily set down to 1, and all other gene expression levels were normalized to the expression level detected within *Rag1* ^−/−^ mice. Data are combined from three individual experiments (*Rag1* ^−/−^ n = 13; *Rag1* ^−^ *^/^* ^−^ *Irf4* ^−/−^ n = 13). Data were analyzed by Student’s *t* test and are shown as mean ± SEM. ns, not significant. *p < 0.05.

Our results have so far established that IRF4 controls the expansion of transferred T cells and recruitment of a series of putatively pro-inflammatory acting myeloid cells in T cell mediated acute colitis through a T cell-extrinsic mechanism. To gain further insight into the molecular mechanism and tissue micromilieu acting in the colitic tissue on the immune cell network, we performed quantitative gene expression analyses employing a series of markers affiliated to distinct T helper cell subsets (**Figure 3**). As displayed in **Figure 3A**, expression of the prototypical Th1 cytokine IFN-gamma was not differentially expressed between IRF4-competent *vs.* -deficient *Rag1* ^−/−^ T cell recipient mice despite a trend towards reduced expression in the absence of IRF4. However, in contrast, colonic expression levels of IL-17, GM-CSF and TNF-alpha were significantly reduced in the absence of IRF4 compared to controls suggesting hampered representation of a Th17 cell enriched cytokine milieu *in situ*. To specifically assess whether Th17 cell differentiation inducing and/or Th17 cell promoting cytokines are differentially regulated, we compared colonic gene expression profiles of IL-12 (Th1) and IL-1*ß*/IL-6/IL-23 (Th17) between colitis-protected *Rag1* ^−/−^ *Irf4* ^−/−^ and colitic IRF4-competent mice (**Figure 3B**). With the exception of *Il23a*, *i.e.* IL-23p19 the colonic tissue expression of all molecules was significantly reduced in the absence of IRF4 clearly indicating that IRF4 expressed by non-T cells might be critical for the generation of a Th17 prone microenvironment. In line with a predominately Th17 differentiation permitting tissue micromilieu, additional quantification of total IL-23R expression revealed significantly upregulated tissue levels in the presence of T cell extrinsically expressed IRF4 (**Figure 3B**). Overall enhanced IL-23R expression might indicate increased susceptibility of invading immune cells including T cells to be permissive for IL-23 mediated effects as Th17 cell differentiation and/or expansion. Since our data so far clearly suggested that molecules and pathways feeding into Th17 cell rather than Th1 cell differentiation are positively regulated by IRF4 through a T cell-extrinsic mechanism, we finally assessed tissue expression levels of transcription factors firmly connected to Th1 and Th17 differentiation, respectively (**Figure 3C**). Interestingly, tissue T-bet/*Tbx21* expression widely accepted to be a critical regulator of Th1 differentiation was virtually indistinguishable between both *Rag1* ^−/−^ cohorts irrespective of their ability to express IRF4 or not (48). In contrast, however, and in full agreement with the data presented above (**Figures 3A, B** **)**, *Rag1* ^−/−^ mice deficient in IRF4 showed a significantly reduced tissue expression of *bona fide* Th17 regulating transcription factors ROR*γ*t and BATF (39, 49). Collectively, our data demonstrate that IRF4 expression by non-T cells in T cell receiving *Rag1* ^−/−^ mice is crucial for the establishment of a Th17 prone microenvironment in the colon suggesting that IRF4 may control T cell mediated colitis through provision of Th17 cell differentiation enabling and promoting mechanisms outside of T cells.

### IRF4 Is Indispensable for the Formation, Expansion, and Intestinal Homing of Th17 but Not Th1 Cells in a T Cell-Extrinsic Manner

**Figure 4:**
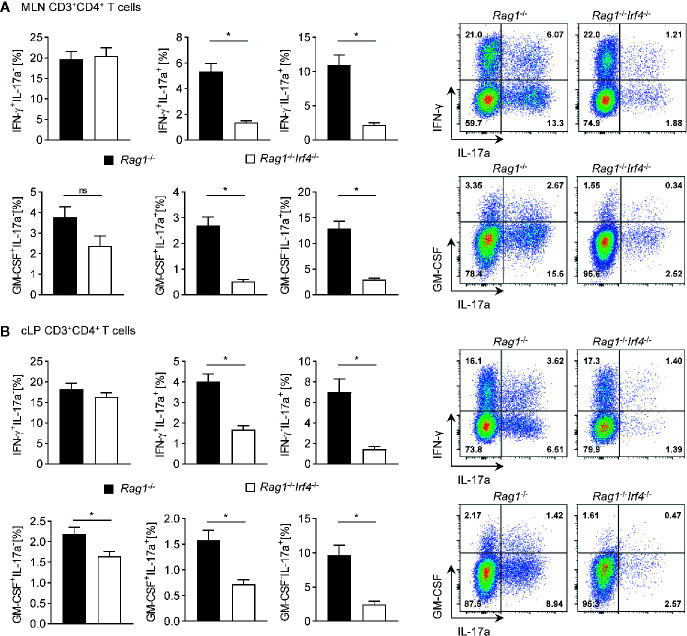
Multiple Th17 cell subsets but not *bona fide* Th1 cells are critically dependent on the expression of functional IRF4 in *Rag1* ^−/−^ mice with established T cell driven colitis. *Rag1* ^−/−^ and *Rag1* ^−^ *^/^* ^−^ *Irf4* ^−/−^ mice were injected i.p. with 1 × 10^6^ naïve (CD4^+^CD25^−^) T cells. When colitis was established in *Rag1* ^−/−^ mice five to six weeks after T cell transfer, both *Rag1* ^−/−^ and *Rag1* ^−^ *^/^* ^−^ *Irf4* ^−/−^ mice were sacrificed, and the cytokine profile of transferred CD4^+^ T cells (CD3^+^CD4^+^) in the MLN **(A)** and in the cLP **(B)** was analyzed by intracellular flow cytometry after *ex vivo* restimulation. Frequencies of IFN-*γ* ^+^IL-17a^−^, IFN-*γ* ^+^IL-17a^+^, IFN-*γ* ^−^IL-17a^+^ (upper panel) and GM-CSF^+^IL-17a^-^ GM-CSF^+^IL-17a^+^ GM-CSF^−^IL-17a^+^ (lower panel) expressing cell populations within the CD4^+^ T cell pool were analyzed. One representative flow cytometry plot is shown for every experimental group. Data are combined from two individual experiments (*Rag1* ^−/−^ n = 7; *Rag1* ^−^ *^/^* ^−^ *Irf4* ^−/−^ n = 8). Data were analyzed by Student’s *t* test and are shown as mean ± SEM. ns, not significant. *p < 0.05.

To further test our hypothesis that missing IRF4 expression within non-T cells results in the abrogated formation of colitogenic Th17 cells and that this step is critical as it may mainly account for alleviated colitis manifestation in this group, we performed in-depth intracellular cytokine staining profiling experiments employing flow cytometry (**Figure 4**). To achieve this goal, following *ex vivo* restimulation we stained both MLN and cLP derived CD4^+^ T cells for IFN-gamma, IL-17a, and GM-CSF expression and compared frequencies of Th1 and Th17 subsets resp. between *Rag1* ^−/−^ *Irf4* ^−/−^ and IRF4-sufficient control mice. In accordance with our gene expression profiling experiments, T-bet dependent Th1 cells defined as IFN-gamma^+^IL-17a^−^ T cells were detected irrespective of the presence or absence of IRF4 expression and hence appear to develop independent of T cell-extrinsic IRF4 expression (**Figures 4A, B** **)**. In sharp contrast, however, IL-17a producing T cell subsets including IL-17a single producing as well as IL-17a^+^ T cells co-expressing either IFN-gamma or GM-CSF were broadly negatively affected in mice lacking T cell-extrinsic IRF4 expression (**Figures 4A, B** **)**. Strikingly, the comparison of both MLN and cLP displayed an unequivocal pattern across organs in the absence of T cell-extrinsic IRF4 expression overall supporting the conclusion that reduced Th17 cell fractions in these mice reflect a rather general negative effect on Th17 cell differentiation and eventually Th17 cell pool than merely results from hampered colonic influx of in MLN otherwise appropriately skewed and functionally equipped Th17 cells.

Finally, to shed light on the mechanism putatively underlying altered T cell instruction and proliferation in the absence of IRF4, we screened for IRF4-dependent changes in the colonic innate immune cell pool in the steady state, *i.e.* prior T cell transfer. In regard to innate lymphoid cells (ILC) with presumably overall rather colitis-suppressive effects given the fact that ILC depletion leads to enhanced colitis manifestation (50, 51), we found that the colonic pool of ILC1, ILC2, and ILC3 forms in a virtually IRF4 expression independent manner **(** **Supplementary Figure 2** **)**. Next, we assessed the antigen-presenting cell (APC) compartment in *Rag1* ^−^ *^/^* ^−^ *vs. Rag1* ^−/−^ *Irf4* ^−/−^ mice **(** **Supplementary Figure 3** **)**. In support of the possibility that IRF4 deficient APCs may primarily account for the observed reduced ability to skew transferred naïve T cells into colitogenic Th17 cells, we confirmed data from previous reports (15, 16, 24) by demonstrating that the pool of Th17 responses promoting cDC2s but not cDC1s is reduced in the absence of IRF4 both in the spleen and MLN (**Supplementary Figures 3A, B** **)**. Although, colonic DC populations were not regulated in the absence of IRF4 **(** **Supplementary Figure 3C** **)**, flow cytometric expression profiling among colonic APCs interestingly revealed that predominately CD11b^+^ cDCs, *i.e.* cDC2s regulated in both MLN and spleen, express higher levels IRF4 protein compared to CD103^+^CD11b^−^ cDC1 **(** **Supplementary Figure 3D** **).** This result implies that albeit not diminished in numbers, colonic cDC2 might be functionally affected (*e.g.* migratory abilities) upon IRF4 deletion and at least partially contribute to compromised induction of colitogenicity within transferred T cells.

Collectively, given the hampered colitis formation in T cell recipient *Rag1* ^−/−^ mice lacking IRF4, here we provide data giving crucial mechanistic insight into the molecular mechanisms by showing that IRF4 controls the formation of colitogenic BATF- and ROR*γ*t-dependent Th17 cell subsets in a T cell-extrinsic manner.

## Discussion

Clinical practice in IBD management has profoundly changed upon the availability of targeted therapies. While antibody-mediated blockade of the cytokine TNF-alpha represents a mainstay in the management of chronic inflammatory diseases including IBD, the expression of additional cytokines as *e.g.* IL-1ß, IL-6, IL-17, and IL-23 has been identified to be highly upregulated within inflamed tissues (7, 52–54). While clinical studies demonstrated that inhibition of some of those cytokines is effective at least in certain disease entities, in IBD only IL-23 targeting showed convincing inflammation-reducing effects and hence was approved for this indication (55). Molecularly, IL-23 employs multiple mechanism thereby affecting a series of immune cells to promote immune-mediated tissue inflammation (10–12, 56–58). However, IL-23 is predominately fostering pro-inflammatory T helper cells that share a common feature, *i.e.* the expression of IL-17a (10, 59). Hence, the identification of alternative targeting strategies going beyond IL-23 neutralization to contain Th17 cell driven tissue destruction remains a valuable goal for basic research enterprises. Recently, effective targeting of the transcriptional regulator GATA3, which is being widely accepted to promote type 2 tissue immune responses by regulating gene expression on a transcriptional level in multiple immune cell subsets implied in the pathogenesis of allergic airway diseases, was reported to be achievable by local application of GATA-3-specific DNAzymes, thereby showing clinical efficacy in asthma patients (60, 61). Hence, targeting transcription factors to tackle mucosal inflammation may represent a challenging but putatively rewarding research area also in the context of Th17 mediated tissue inflammation. In the chain of events underlying the initiation of an inflammation cascade, transcription factors act rather upstream and thereby often control pathogenetically and functionally related networks acting across cell type borders. The identification and interference with a potential master switch within the Th17 network would hence represent a major advance for the field of targeted immunotherapy of immune-mediated chronic inflammatory disorders.

Until know, T cell-intrinsic expression of a series of transcriptional regulators like BATF, ROR*γ*t and IRF4 has been identified to be crucial for a T cell to differentiate into a pro-inflammatory Th17 cell (39, 49, 62). Interestingly, transcription factors usually regulate multiple milestones within a given cell type both by direct and indirect transcriptional effects. For example, besides regulating the expression of IL-17 cytokine family members directly, BATF is indispensable for continuous ROR*γ*t expression and hence controls through this regulation of the Th17 cell network also indirectly the expression of the IL-23 receptor in T cells (39, 63, 64). Among Th17 fate regulating transcription factors, especially IRF4 seems to exert crucial functions in a series of T cell-extrinsic immune cell populations including innate lymphoid cells (ILCs), dendritic cells, and monocytes (16, 19, 65–67). However, the functional role of IRF4 expressed in non-T cells in the context of intestinal inflammation has not gained much attention. We found here in this study that inactivation of IRF4 employing germ line deletion disables IRF4-proficient T cells to mediate disease in a widely accepted T cell dependent mouse model of acute colitis. Mechanistically, our analyses revealed that the establishment of a Th17 inducing cytokine milieu in the colon, as *e.g.* upregulated IL-1ß and IL-6 expression, required IRF4 while for IL-23 expression it was not. While the reduction of IL-1ß and IL-6 might be sufficient to explain hampered Th17 differentiation, the missing regulation of IL-23 expression in colitic tissue appears at first sight puzzling for two reasons: First, IRF4 dependent cDC2s have been described to be the major producer of IL-23 among DCs and thereby putatively impacting Th17 cell differentiation directly (16, 19). Second, to account for the hampered proliferation of differentiated Th17 cells as one possible explanation for the reduced colitis manifestation appears at first sight rather unlikely given the widely accepted dominant role of IL-23 in this context through the provision of critical survival and expansion signals especially for IL-23 receptor expressing Th17 cells (12, 13). However, although we have not tested that possibility directly, in the light of downregulated BATF/ROR*γ* t both known to regulate IL-23 receptor expression T cells primed in IRF4-deficient *Rag1* ^−^ *^/^* ^−^ mice in fact may lack IL-23R expression on their cell surface. Thereby T cells were unable to receive proliferation-promoting effects from IL-23, overall providing an explanation for their mitigated colitogenicity. In this scenario and supported by our data, colonic IL-23 levels are mounted by cells functioning at least in this respect independent of IRF4, resulting in similar total IL-23 tissue levels. In fact, T cells selectively lacking IL-23R through genetic inactivation similarly fail to mediate colitis in this model system (12). Hence, despite indistinguishable provision of IL-23, effects dependent on the IL-23/IL-23R interaction on T cells appear to be extinguished in the absence of IRF4 in non-T cells due to virtually absent IL-23R expression on developing effector T cells. In fact, IL-23R expression analysis within total colon tissues revealed strikingly reduced levels presumably due to decreased total T cell numbers in the colon but most likely also reflecting reduced IL-23 receptor copies expressed by a single cLP T cell. Future studies certainly need to further investigate the question which molecular signals derived from the remaining and putatively functionally compromised IRF4-deficient cDC2s may directly or indirectly account for proper induction of IL-23 receptor expressing, colitogenic Th17 cells. Interestingly, IL-6 was shown to induce both BATF and ROR*γ*t in a STAT3-dependent manner (42). Given reduced IL-6 tissue levels, IL-6 expression might be dependent on the presence of IRF4 within myeloid cells both in the MLN and/or cLP (16, 19). Hence, IRF4 deficiency-related reduction of IL-6 expression might additionally undermine Th17 differentiation. This scenario of IL-6 driven Th17 cell differentiation may be clinically relevant given the clinical observation that some patients do not respond to IL-23 blockade despite a Th17 dominated mucosal inflammation *in situ*. However, this hypothesis will require vigorous testing in the future especially in the light of rather moderate results that studies investigating the efficacy of an IL-6 blocking antibody (tocilizumab) in IBD reported (68, 69).

Hence, based on our current study, global interference with IRF4 expression and consecutively IRF4-dependent pathways might emerge as a promising therapeutic option gain control especially in those sub-cohorts of IBD patients suffering from continuous intestinal inflammation refractory to all currently available and clinically approved treatment regimens. Due to its documented, dual colitogenic role both in T cells and non-T cells, IRF4 targeting may be in this regard a very attractive molecular target to limit intestinal inflammation by inhibiting both development and functionality of Th17 cells.

Since the identification of the precise IRF4-dependent non-T cell immune cell(s) and/or signaling pathways putatively regulating T cell driven colitis was not the focus of this study and in fact will require already planned future experimentation, we can only speculate on that at this point. Although IRF4 has been clearly shown to impact ILC biology (65), antibody mediated ablation of ILC in fact leads to an aggravation of colitis (data not shown) and as previously published (50, 51) indicating that hampered ILC functionality is not likely to underlie the colitis protection observed in the absence of IRF4. In addition, in this study we provide experimental data that IRF4 deficiency does not alter the pool of colonic ILC1, ILC2 or ILC3 on Rag1^−/−^ background. While this does not formally exclude an ILC-mediated mechanism underlying colitis protection in the absence of IRF4, our and published data, however, are not in favor of the conclusion that regulation of ILC function is here involved. Based on the currently available literature, among myeloid cells known to express IRF4 the functionality of APC may appear to be most likely regulated in an IRF4-dependent manner and by this control colitogenic T cell differentiation (15, 16, 44). In CD11b^+^ cDCs, *i.e.* cDC2, IRF4 regulates migration to (70) and survival within mucosal tissues (16, 19). As elaborated above in respect to the biology of IL-23, cDC2 cells are in part dependent on IRF4 as confirmed in this study and have been put forward to be critical in Th17 cell biology due to its preferential expression of IL-23 among cDCs (16, 19, 71, 72). Future studies including the usage of cDC-specific deletor mice, *i.e.* genetically engineered mice expressing cre recombinase under the control of the promotor of *Zbtb46* crossed to mice carrying a conditionally targeted IRF4 allele will be required to address that question (73, 74). Similarly, distinct functions within monocytes and monocyte-derived cells have been shown to be regulated by IRF4 as *e.g.* the ability to cross-present cell-associated antigens to CD8^+^ T cells (66), fine-tuning TLR signaling (75), cytokine expression (76) or development and functionality of immunosuppressive myeloid cell populations (77). Here, with the help of novel genetic model systems, future studies need to decipher cell-type specific roles of IRF4 within ontogenetically distinct cDC and monocyte-derived cell populations in the context of colitis.

Collectively, our study unequivocally demonstrates that IRF4 expressed in non-T cells is a positive regulator of Th17 cell mediated colitis. Given the T cell-intrinsic role of IRF4 in driving Th17 cell differentiation *in vitro* (62) and also in the context of colitis *in vivo* (32), our data support the conclusion that inactivation of IRF4 universally protects against Th17 mediated colitis and suggest that strategies to interfere with IRF4 gene expression are of potential great interest to harness acute intestinal inflammation.

## Data Availability Statement

The raw data supporting the conclusions of this article will be made available by the authors, without undue reservation.

## Ethics Statement

The animal study was reviewed and approved by government of Lower Franconia.

## Author Contributions

VB, TV, PK, TK, and HK performed the experiments. VB analyzed and interpreted the data together with KH and TV. KE and CN established and performed the immunofluorescence stainings with the help of TV. MH performed the histopathological analyses. CL, LA, SW, and DD gave important advice and provided crucial reagents. MN gave important advice and helped with the interpretation and critical discussion of the results. KH directed the study and wrote the manuscript together with VB and valuable input from all authors. All authors contributed to the article and approved the submitted version.

## Funding

This study was supported by the Collaborative Research Centers 1181 (DFG-CRC1181, project B05, to KH and project A07, to DD), TRR241 (DFG-CRC/TR241 A08 to both CN & KH), TRR221 project B03 to KH, DFG/DU548/5-1 to DD, DFG/HI 849/4-1 and SPP1656/ HI 849/5-1 (both to KH).

## Conflict of Interest

The authors declare that the research was conducted in the absence of any commercial or financial relationships that could be construed as a potential conflict of interest.
