# Supplementary material for: Th17 Cell-Mediated Colitis Is Positively Regulated by Interferon Regulatory Factor 4 in a T Cell-Extrinsic Manner
Source: Front Immunol. 2021 Jan 29;11:590893. doi: 10.3389/fimmu.2020.590893 (PMC7879684; doi:10.3389/fimmu.2020.590893)
Supplement: Supplementary file 1 [file DataSheet_1.docx]

Supplementary Material


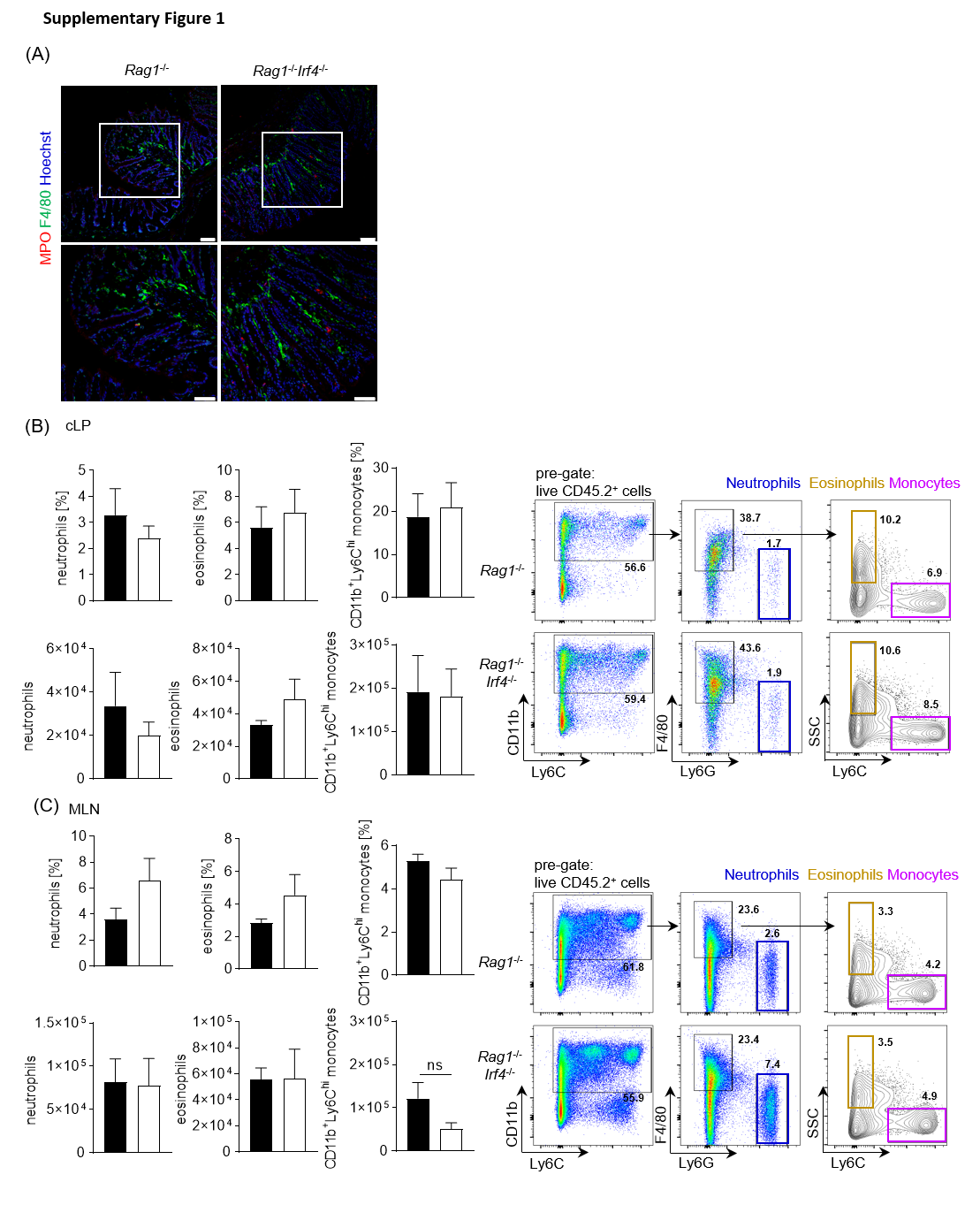


**Supplementary Figure 1.** **IRF4 deficiency on a Rag1^-/-^ background does not affect the cellular composition of MLN and cLP resident granulocyte subsets and Ly6C^high^ monocytes in the absence of inflammation.** (A) Representative immunofluorescence staining of F4/80^+^ and MPO^+^ cells in colonic cross sections of *Rag1*^-/-^ vs. *Rag1^-/-^Irf4*^-/-^mice. *Upper panel*: lower magnification image of the colonic tissue (scale bars = 75 µm), white box indicates the area in the colon section that is displayed in the *lower panel* with a higher magnification (scale bars = 50 µm). (B-C) Relative fraction (upper panels) and absolute cellularity (lower panels) of neutrophils (CD11b^+^F4/80^-^Ly6G^+^Ly6C^+^), eosinophils (CD11b^+^F4/80^+^Ly6G^-^SSC^high^Ly6C^-^) and inflammatory Ly6C^high^ monocytes (CD11b^+^F4/80^+^Ly6G^-^SSC^low^Ly6C^high^) within the total live CD45.2^+^ immune cell pool of cLP (B) and MLN (C) cells were assessed by flow cytometry. The right panels of (B) and (C) display one representative flow cytometry plot for each genotype and anatomic location to illustrate the gating strategy for the indicated cell populations. Cell frequencies in each sub-gate are calculated as a percentage of total live CD45.2^+^ cells. Data are from one experiment (*Rag1*^-/-^ n = 5; *Rag1^-/-^Irf4*^-/-^ n = 5). Data were analyzed by Student’s *t* test and are shown as mean ± SEM. ns = not significant.


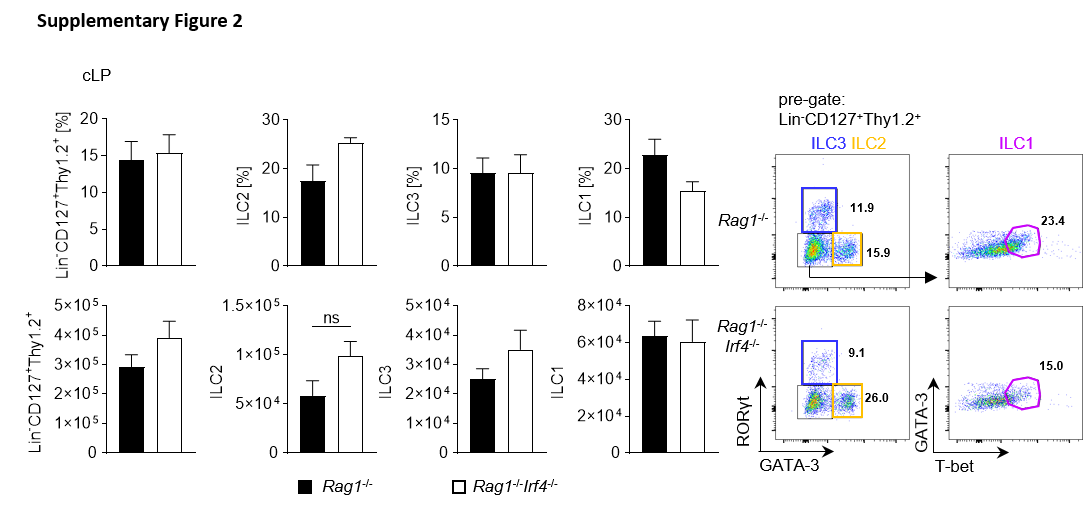


**Supplementary Figure 2.** **No significant alteration of the colonic ILC pool in *Rag1^-/-^Irf4^-/-^*** **compared to *Rag1*^-/-^ mice in the steady state.** The frequency (%) of total ILCs (Lin^-^CD127^+^Thy1.2^+^) within the total live cell pool and of ILC1 (Lin^-^CD127^+^Thy1.2^+^GATA3^-^RORγt^-^T-bet^+^), ILC2 (Lin^-^CD127^+^Thy1.2^+^GATA3^+^), and ILC3 (Lin^-^CD127^+^Thy1.2^+^RORγt^+^) subsets within total Lin^-^CD127^+^Thy1.2^+^ ILCs within the cLP were determined by flow cytometry. Shown frequencies for individual ILC subsets reflect the representation of the cells within each sub-gate relative to the bulk ILC population. In addition, total cell numbers of indicated ILC subsets were calculated. A representative flow cytometry plot is displayed illustrating the gating strategy to identify and define indicated ILC subsets. cLP data (*Rag1*^-/-^ n = 8; *Rag1^-/-^Irf4*^-/-^ n = 6) represent pooled data from two independent experiments. Data were analyzed by Student’s *t* test and are shown as mean ± SEM. ns = not significant.


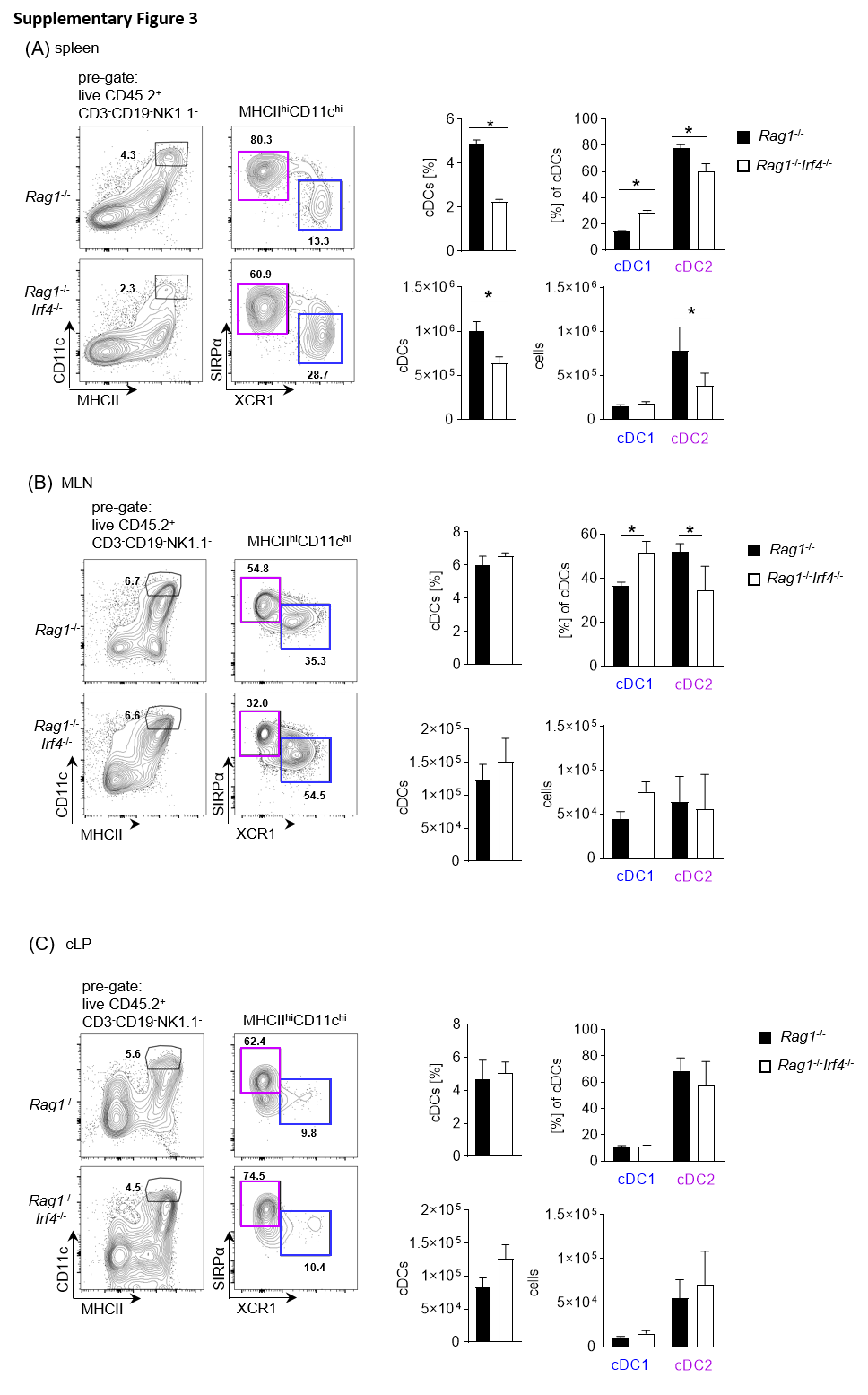

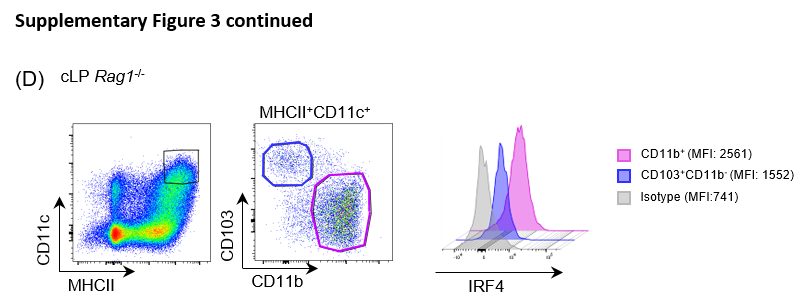


**Supplementary Figure 3. IRF4 deficiency reduces cDC2s in spleen and MLN**. The frequency (%) and absolute number of the indicated DC populations in spleen (A), MLN (B), and cLP (C) of untreated *Rag1*^-/-^ and *Rag1^-/-^Irf4*^-/-^ mice were analyzed by flow cytometry. cDCs (CD45^+^CD3^-^CD19^-^NK1.1^-^MHCII^hi^CD11c^hi^ cells) were subdivided by the expression of XCR1 or SIRPα/CD172a into cDC1s (XCR1^+^SIRPα^-^; blue) and cDC2s (XCR1^-^SIRPα^+^; purple). The frequency of cDCs within the total live cell pool and the frequency of cDC1 and cDC2 within cDCs are shown. A representative flow cytometry plot is displayed depicting the gating strategy to identify and define indicated DC subsets. cLP (*Rag1*^-/-^ n = 7; *Rag1^-/-^Irf4*^-/-^ n = 8) and spleen (*Rag1*^-/-^ n = 12; *Rag1^-/-^Irf4*^-/-^ n = 10) data represent pooled data from two independent experiments and MLN (*Rag1*^-/-^ n = 4; *Rag1^-/-^Irf4*^-/-^ n = 3) data represent data from one experiment. Data were analyzed by Student’s *t* test and are shown as mean ± SEM. **p* <0.05. (D) Intracellular IRF4 protein expression within MHCII^hi^CD11c^hi^ cLP cells was determined by flow cytometry. Gating strategy to identify CD11b^+^ DCs, i.e. cDC2s, and CD103^+^ CD11b^-^ cDC1s in the cLP of *Rag1*^-/-^ mice is shown. The IRF4 expression within these populations is displayed as a histogram and represents mean fluorescence intensity (MFI) compared to an isotype control antibody.
